# Hip circumference has independent association with the risk of hyperuricemia in middle-aged but not in older male patients with type 2 diabetes mellitus

**DOI:** 10.1186/s12937-023-00874-5

**Published:** 2023-09-22

**Authors:** Wenyi Lu, Xuan Zhao, Jinye Sheng, Xuelin Zhao, Qingya Tang, Hongmei Zhang, Yi Feng, Yang Niu

**Affiliations:** 1 Department of Clinical Nutrition, Xinhua Hospital Affiliated to Shanghai Jiao Tong University School of Medicine, Shanghai, 200092 China; 2 Department of Endocrinology, Xinhua Hospital Affiliated to Shanghai Jiao Tong University School of Medicine, Shanghai, 200092 China; 3 Department of Clinical Nutrition, College of Health Science and Technology, Shanghai JiaoTong University School of Medicine, Shanghai, China

**Keywords:** Type 2 diabetes, Hyperuricemia, Obesity, hip circumference, Middle-aged and older male patients

## Abstract

**Background:**

Obesity and type 2 diabetes mellitus (T2DM) are risk factors for hyperuricemia. However, which anthropometric indices can better predict incident hyperuricemia in patients with T2DM remains inconsistent. This study aimed to examine the associations between hyperuricemia and different anthropometric indices in middle-aged and older male patients with T2DM.

**Methods:**

In this retrospective study, a total of 1447 middle-aged (45—65 years, *n* = 791) and older (≥ 65 years, *n* = 656) male patients with T2DM were collected from December 2015 to January 2020 at Shanghai Xinhua Hospital. Hyperuricemia was defined as a serum uric acid level above 7.0 mg/dL. Weight, height, waist circumference (WC) and hip circumference (HC) were measured by trained nurses at visit.

**Results:**

The median uric acid level of subjects was 5.6 (interquartile ranges: 4.7—6.7) mg/dl, and 279 (19.3%) were hyperuricemia, with 146 (18.5%) in the middle-aged group, and 133 (20.3%) in the older group. After adjusting for age, duration of T2DM, fasting plasma glucose and insulin, homeostasis model assessment-β, aspartate aminotransferase, triglycerides, high-density lipoprotein cholesterol and estimated glomerular filtration rate, body mass index (BMI), WC, HC, and waist-to-height ratio (WHtR) were associated with a higher risk of hyperuricemia in both middle-aged and older group (*P* < 0.05). After further adjusting for BMI and WC, HC still showed a positive relationship with the risk of hyperuricemia (Odds Ratio = 1.51, 95% confidence intervals: 1.06—2.14) in the middle-aged group, but such relationship was not found in the older group. Moreover, according to receiver operating characteristic analysis, the optimal cutoff value was 101.3 cm of HC for hyperuricemia screening in the middle-aged male patients with T2DM.

**Conclusion:**

In middle-aged male patients with T2DM, more attention should be paid to HC with the cutoff value of 101.3 cm in clinical practice for early recognition of individuals with a high risk of hyperuricemia for targeted guidance on disease prevention, such as community screening.

## Introduction

Type 2 diabetes mellitus (T2DM) is considered as a public health problem worldwide. 537 million adults are living with diabetes, according to the latest report in the International Diabetes Federation Diabetes Atlas [1]. Over the past three decades, the prevalence of diabetes in China has a sharp increase, resulting in its being the most populous country with an estimated 109.6 million adults with diabetes [2]. Accumulated studies have demonstrated that hyperuricemia (HUA), defined as the presence of an increased serum uric acid (UA) concentration, is common in patients with T2DM.

UA is a metabolite derived from the oxidation of purine compounds, and it is also an important biological regulator. Although the previous study showed no evidence of clinically meaningful benefits of serum urate reduction on kidney outcomes among patients with early-tomoderate diabetic kidney disease [3]. The level of UA exceeding the physiological level has been suggested as an important role in the development of metabolic diseases and cardiovascular disease. One prospective cohort study found that HUA was positively associated with all-cause mortality and cardiovascular disease mortality in patients with diabetes [4]. Another prospective cohort study of 123,238 Chinese patients showed that participants with the highest quintile of serum urate had 1.91‐fold higher risk of atrial fibrillation [5]. HUA is also an independent risk factor for hypertension [6]. Some studies suggested that the pathogenesis is related to the redox-dependent signaling and oxidative stress induced by HUA in adipocytes, and oxidative stress in the adipose tissue has been recognized as a major cause of insulin resistance and cardiovascular disease [7]. Therefore, it is important to find out a simple and rapid detection method for early recognition of individuals with high risk of HUA for targeted guidance on disease prevention, such as community screening.

Although growing evidence has found that HUA is associated with obesity and body mass index (BMI) [8–10], the role of body fat distribution in UA metabolism is still ambiguous. BMI as an overall evaluation of general obesity, cannot distinguish between muscle and fat accumulation, which means it cannot comprehensively assess the metabolic differences. And several studies have shown that lower body circumference is inversely associated with metabolic diseases, such as diabetes, cardiovascular diseases, and total mortality, unlike upper body circumference [11–14]. Some studies suggested that the pattern of body fat distribution is a more important determinant of disease risk than BMI. A standardized case–control study of acute myocardial infarction showed that the population-attributable risks of myocardial infarction for increased waist-to-hip ratio (WHR) in the top two quintiles was 24.3% compared with only 7.7% for the top two quintiles of BMI [15]. In another study of 2,683 post-menopausal women, after adjustment for cardiovascular risk factors, both elevated trunk fat and reduced leg fat were associated with increased risk of cardiovascular disease, whereas neither whole-body fat mass nor fat percentage showed significant associations [16]. Due to the advantages of convenience, simplicity, harmlessness, and low cost, anthropometric measurements, especially circumferences, are useful options for estimating fat distribution and body composition, particularly in large-scale population-based studies.

Accordingly, this study aimed to examine the relationship between different anthropometric indices, including BMI, waist circumference (WC), hip circumference (HC), WHR, and waist-to-height ratio (WHtR), and the risk of HUA in patients with T2DM. Furthermore, considering the age- and gender-specific differences in the prevalence and risk factors of hyperuricemia have been reported [17–19], the current study focused on the middle-aged and older male subjects.

## Materials and methods

### Study population

**Fig. 1:**
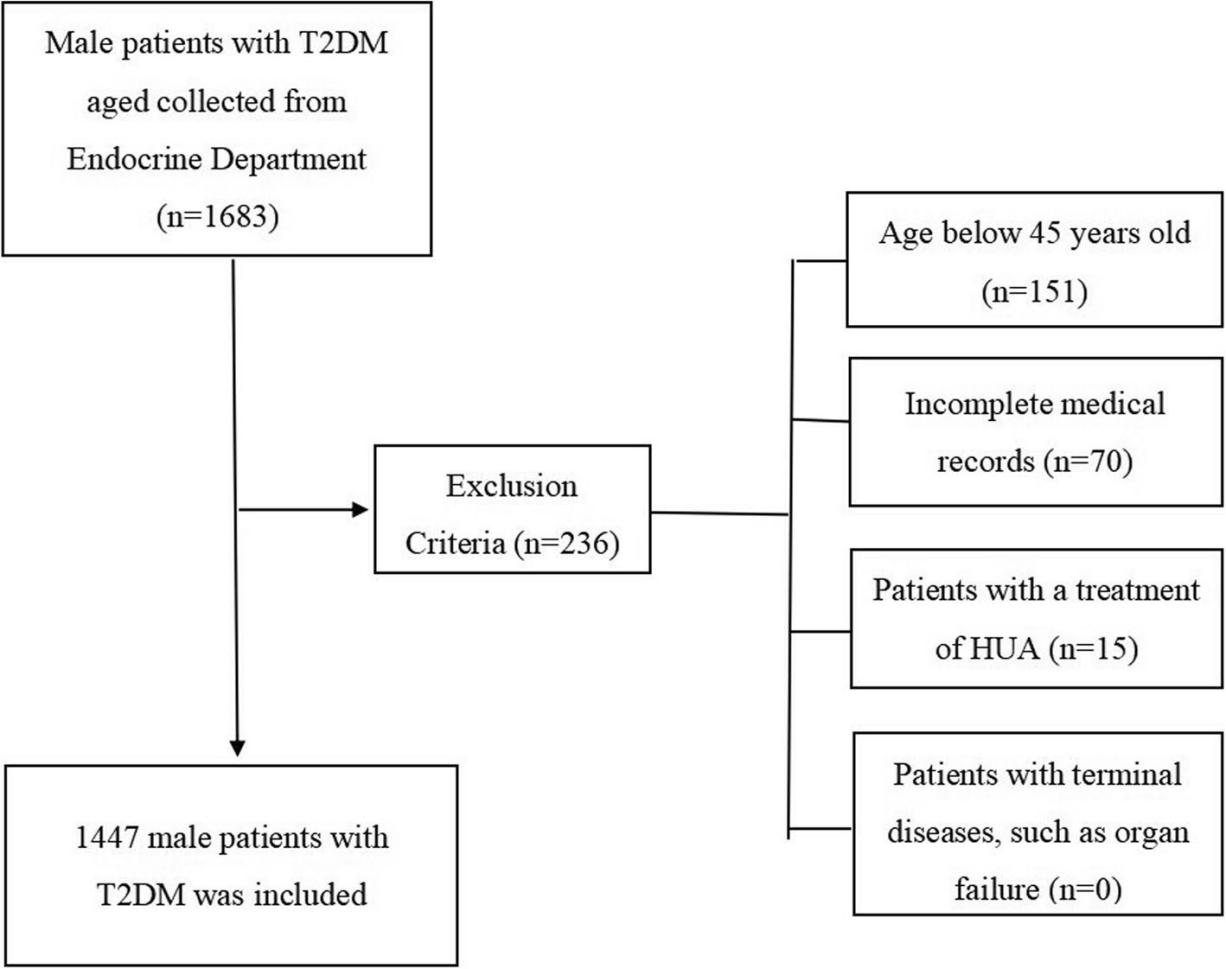
Flow chart of collecting data. Abbreviation: T2DM, type 2 diabetes mellitus; HUA, hyperuricemia

A retrospective cross-sectional study was conducted at Xinhua Hospital, School of Medicine, Shanghai Jiao Tong University. Male patients with T2DM, diagnosed according to American Diabetes Association 2013 criteria [20], aged 45 years and above were included, from December 2015 to January 2020. The current study excludes patients with a treatment of HUA, missing data of anthropometric indices, and patients with terminal diseases, such as organ failure (Fig. 1). This study was approved by the Ethics Committee of Xinhua Hospital affiliated to Shanghai Jiao Tong University School of Medicine (No. XHEC-D-2022–091).

### Data collections

#### Anthropometric assessments

Weight and height were measured by trained nurses at visit with indoor clothing and without shoes. WC and HC were measured using a measuring tape in a standing position. For the WC measurement, the tape was wrapped at mid-height between the lower edge of the last rib and the upper edge of the iliac crest. And HC was measured by applying the measuring tape at the most prominent part of the buttocks. All measurements were performed in duplicate and averaged. All anthropometric indices were recorded to the nearest 0.1 cm or kg.

WHR and WHtR were calculated as the WC (cm) divided by the HC (cm) and height (cm), respectively.

#### Definition of HUA

HUA was defined as a serum UA level above 7.0 mg/dL [21]. Subjects were divided into HUA group (UA ≥ 7.0 mg/dL) and non-HUA group (UA < 7.0 mg/dL) according to the serum UA level. And UA was measured in overnight fasting state and detected by the uricase method using a Hitachi 7600 automatic biochemical analyzer (Hitachi, Tokyo, Japan).

#### Covariates

The essential information was collected from hospital electronic records, including sex, age and previous medical history. Blood Pressure (BP) was measured with an automated electronic device (Omron J710; Omron Company, Kyoto, Japan) after the subjects sat down and rested quietly for > 5 min. Peripheral venous blood samples were collected after 10 h of overnight fasting and sent to the Clinical Laboratory Center for laboratory test. Serum concentrations of creatinine, triglycerides (TG), high-density lipoprotein cholesterol (HDL-C), low-density lipoprotein cholesterol (LDL-C) and total cholesterol (TC) were measured by an enzymatic colorimetric assay (Type 7600; Hitachi, Tokyo, Japan). Fasting plasma glucose (FPG), alanine aminotransferase (ALT) and aspartate aminotransferase (AST) levels were measured by using an automated analyzer. Fasting insulin (FINS) was estimated by chemiluminescence method (Abbott ARCHITECT i2000R, Chicago, USA).

Homeostasis model assessment (HOMA) models were used to estimate insulin resistance (HOMA-IR) and β-cell function (HOMA-β, %), which were calculated by the following simplified equations: HOMA-IR = (FINS × FPG) / 22.5, and HOMA-β = (20 × FINS) / (FPG—3.5) [22, 23]. The value of estimated glomerular filtration rate (eGFR) was calculated with the following equation: eGFR (mL/min/1.37m^2^) = 175 × creatinine^−1.234^ × age^−0.179^, where creatinine is in mg/dl and age is in years [24].

#### Statistical analysis

The characteristics of patients were presented as the means ± standard deviation (SD) or medians (interquartile ranges [IQR]). For the categorical variables, they were presented as numbers and percentages (%). Comparisons among the difference between HUA group and non-HUA group were conducted using the independent two-tailed *t*-tests or Wilcoxon signed-rank tests. The association between potential covariates and UA levels was assessed through Spearman correlation analysis. Logistic regression models were used to evaluate the impacts of different anthropometric indices on the risk of HUA, including BMI, each SD increase in WC, HC, WHR, and WHtR.

Baseline covariates with *P* < 0.05 from the univariate analysis were subsequently included stepwise logistic regression analysis, and odds ratios (OR) and 95% confidence intervals (CI) were calculated. In further multiple logistic regression analysis, Model 1 was an unadjusted regression; Model 2 was a regression with FPG, FINS, HOMA-β, AST, TG, HDL-C and eGFR adjusted; Model 3 was a regression with BMI adjusted building upon Model 2; Model 4 was a regression with WC adjusted building upon Model 3.

Because of the independent association between HC and HUA shown in the full adjusted model in the middle-aged group, we further performed the receiver operating characteristic (ROC) analysis and the area under ROC curves (AUC) to evaluate the predictive power and optimal cutoff value of HC for identifying HUA in this age group. The optimal cutoff value was determined by the maximized Youden index value (Sensitivity + Specificity—1).

All analyses were performed using IBM SPSS Statistics version 25 (IBM Corp., Armonk, NY, USA). Two-sided *P* value < 0.05 was considered statistically significant.

## Results

### Characteristics of subjects

A total of 1447 subjects aged 45 to 91 years old were included in this study, 791 (54.7%) being middle-aged (aged 45—64 years old) and 656 (45.3%) being older (aged 65 years old and above). The median UA level was 5.6 (IQR: 4.7—6.7) mg/dl. HUA was observed in 279 (19.3%) of the study population. The prevalence of HUA was no statistical difference between the middle-aged group and the older group (18.5% vs. 20.3%, respectively; *P* = 0.383).

Table 1 summarized the characteristics of study subjects between the HUA and non-HUA groups. Subjects in the HUA group were more likely to be with a higher FINS, HOMA-β, TG, and a lower HDL-C and eGFR level than those in the non-HUA group in both middle-aged group and older group (all *P* < 0.05). In addition, the HUA group had a longer duration of T2DM and a lower FPG in the middle-aged group (all *P* < 0.05). Compared with non-HUA subjects, BMI, WC, HC, and WHtR of subjects with HUA were higher in both middle-aged and older group (all *P* < 0.05).

**Table 1:** Characteristics of study subjects

Variable	Total (*n* = 1447)		Middle-Aged Group (*n* = 791)		Older Group (*n* = 656)	
	**HUA (*n* = 279)**	**Non-HUA (*n* = 1168)**	**HUA (*n* = 146)**	**Non-HUA (*n* = 645)**	**HUA (*n* = 133)**	**Non-HUA (*n* = 523)**
Age, y	64.0 (59.0–69.0)	63.0 (58.0–69.0)	59.5 (55.0–62.0)	59.0 (55.0–62.0)	69.0 (67.0–73.5)	70.0 (67.0–75.0)
Duration of T2DM, y	11.0 (6.0–19.0)	10.0 (4.0–16.0)^**^	10.0 (5.0–15.3)	9.0 (4.0–13.0)^*^	14.0 (8.0–20.0)	12.0 (5.0–20.0)
BMI, kg/m^2^	25.7 (24.0–27.5)	24.5 (22.5–26.4)^**^	25.8 (24.2–27.8)	24.6 (22.6–26.8)^**^	25.6 (23.6–27.4)	24.4 (22.3–26.1)^**^
WC, cm	96.0 (90.0–100.2)	92.0 (87.0–98.0)^**^	96.0 (89.8–102.0)	92.0 (87.0–98.0)^**^	96.0 (90.0–100.0)	92.0 (87.0–98.0)^*^
HC, cm	100.0 (94.0–104.0)	97.0 (92.0–101.8)^**^	100.0 (94.0–104.3)	97.0 (92.0–101.5)^**^	100.0 (95.0–103.5)	97.0 (92.0–102.0)^*^
WHR	0.96 (0.93–1.00)	0.96 (0.92–0.99)	0.96 (0.93–1.00)	0.96 (0.93–0.99)	0.96 (0.93–0.99)	0.96 (0.91–0.99)^*^
WHtR	0.56 (0.53–0.59)	0.54 (0.51–0.58)^**^	0.55 (0.52–0.59)	0.54 (0.51–0.57)^**^	0.57 (0.53–0.60)	0.54 (0.51–0.58)^*^
UA, mg/dl	7.7 (7.3–8.4)	5.3 (4.5–6.0)^**^	7.8 (7.4–8.5)	5.3 (4.5–6.1)^**^	7.6 (7.2–8.4)	5.3 (4.6–6.0)^**^
FPG, mg/dl	125.8 (106.7–155.1)	136.6 (110.1–172.5)^**^	125.9 (107.1–157.9)	142.0 (113.5–178.6)^**^	123.1 (104.3–152.8)	132.3 (106.8–167.4)
FINS, μIU/mL	9.7 (6.2–15.1)	8.4 (5.3–12.6)^*^	9.3 (5.8–13.9)	8.0 (5.0–12.0)^*^	10.7 (6.4–17.7)	9.1 (5.6- 13.3)^*^
HOMA-IR	3.0 (1.8–5.4)	2.9 (1.7–4.8)	2.9 (1.8–4.5)	2.9 (1.7–4.6)	3.2 (1.9–6.1)	3.0 (1.7–4.9)
HOMA-β	55.0 (34.4–101.0)	41.1 (23.1–75.6)^**^	50.2 (34.0–86.7)	36.7 (22.2–65.3)^**^	59.9 (34.9–130.8)	48.1 (25.0–93.9)^*^
ALT, U/L	19.0 (14.0–29.0)	18.0 (13.0–26.0)	21.0 (15.0–32.0)	19.0 (14.0–28.0)	18.0 (12.0–26.0)	17.0 (13.0–25.0)
AST, U/L	19.0 (16.0–24.0)	18.0 (15.0–23.0)^*^	19.0 (16.0–25.0)	18.0 (15.0–24.0)	20.0 (16.0–24.0)	18.0 (15.0–23.0)
TG, mg/dl	174.3 (123.0–268.1)	123.0 (87.6–183.2)^**^	187.6 (132.5–281.9)	132.7 (94.7–194.7)^**^	155.8 (113.7–238.9)	113.3 (81.4–162.0)^**^
TC, mg/dl	164.9 (135.1–190.3)	160.6 (136.7–188.8)	162.0 (136.7–191.1)	166.8 (141.9–195.4)	169.1 (134.2–188.2)	154.4 (129.3–180.0)
HDL-C, mg/dl	40.2 (35.1–46.3)	43.6 (37.5–51.1)^**^	40.0 (34.4–45.2)	43.6 (37.5–51.0)^**^	40.2 (35.7–47.1)	43.2 (37.5–51.4)^*^
LDL-C, mg/dl	96.5 (75.3–117.4)	92.3 (72.6–115.1)	97.1 (76.1–118.1)	96.9 (76.4–118.0)	96.5 (68.1–116.6)	88.4 (67.2–110.8)
eGFR, mL/min/1.37m^2^	93.5 (72.9–118.2)	119.7 (98.9–142.7)^**^	103.6 (76.7–122.4)	125.6 (106.6–149.7)^**^	87.0 (67.4–107.5)	112.2 (90.6–131.6)^**^
SBP, mm Hg	134.0 (126.0–149.0)	131.0 (125.0–146.0)	133.0 (125.0- 149)	130.0 (123.0–143.0)	135.0 (127.0–150.0)	134.0 (126.0–149.0)
DBP, mm Hg	75.0 (70.0–85.0)	75.0 (70.0–85.0)	77.0 (70.0–87.0)	78.0 (70.0–86.0	72.0 (70.0–83.0)	73.0 (69.0–81.0)

*Abbreviations*: *HUA* Hyperuricemia, *T2DM* Type 2 diabetes mellitus, *BMI* Body mass index, *WC* Waist circumference, *HC* Hip circumference, *WHR* Waist-to-hip ratio, *WHtR* Waist-to-height ratio, *UA* Uric acid, *FPG* Fasting plasma glucose, *FINS* Fasting insulin, *ALT* Alanine aminotransferase, *AST* Aspartate aminotransferase, *TG* Triglycerides, *TC* Total cholesterol, *HDL-C* High-density lipoprotein cholesterol, *LDL-C* Low-density lipoprotein cholesterol, *eGFR* Estimated glomerular filtration rate, *SBP* Systolic blood pressure, *DBP* Diastolic blood pressure

^*^Significance between HUA and non-HUA groups with T2DM (*P* < 0.05)

^**^Significance between HUA and non-HUA groups with T2DM (*P* < 0.001)

### Different anthropometric indices and the risk of hyperuricemia

As can be seen in Table 2, BMI, WC, HC, WHR and WHtR showed positive correlations with UA levels (*r* = 0.224, 0.184, 0.153, 0.129, 0.183, respectively; all *P* < 0.001), although there was no significant difference in WHR between HUA and non-HUA groups in the total subjects (median [IQR], 0.96 [0.93—1.00] vs. 0.96 [0.92—0.99], *P* > 0.05).

**Table 2:** Correlation analysis of potential covariates associated with UA levels

	***R***^**a**^	*P* value ^*^
Age	-0.001	0.956
Duration of T2DM	0.077	0.003
BMI	0.224	< 0.001
WC	0.184	< 0.001
HC	0.153	< 0.001
WHR	0.129	< 0.001
WHtR	0.183	< 0.001
FPG	-0.125	< 0.001
FINS	0.124	< 0.001
HOMA-IR	0.057	0.031
HOMA-β	0.168	< 0.001
ALT	0.054	0.04
AST	0.070	0.007
TG	0.307	< 0.001
TC	0.044	0.094
HDL-C	-0.188	< 0.001
LDL-C	0.047	0.074
eGFR	-0.385	< 0.001
SBP	0.055	0.036
DBP	0.003	0.914

*Abbreviations*: *UA* Uric acid, *T2DM* Type 2 diabetes mellitus, *BMI* Body mass index, *WC* Waist circumference, *HC* Hip circumference, *WHR* Waist-to-hip ratio, *WHtR* Waist-to-height ratio, *UA* Uric acid, *FPG* Fasting plasma glucose, *FINS* Fasting insulin, *ALT* Alanine aminotransferase, *AST* Aspartate aminotransferase, *TG* Triglycerides, *TC* Total cholesterol, *HDL-C* High-density lipoprotein cholesterol, *LDL-C* Low-density lipoprotein cholesterol, *eGFR* Estimated glomerular filtration rate, *SBP* Systolic blood pressure, *DBP* Diastolic blood pressure

^a^*r* = Spearman’s correlation coefficient

^*^P values are two-tailed. Values are unadjusted

In the middle-aged group (Table 3), logistic regression results showed that higher BMI (Model 1: OR = 1.10, 95%CI: 1.05—1.17; Model 2: OR = 1.07, 95%CI: 1.01—1.14), and each SD increase in WC (Model 1: OR = 1.39, 95%CI: 1.16—1.65; Model 2: OR = 1.27, 95%CI: 1.04—1.56), HC (Model 1: OR = 1.48, 95%CI: 1.23—1.78; Model 2: OR = 1.40, 95%CI: 1.14—1.72) and WHtR (Model 1: OR = 1.36, 95%CI: 1.14—1.63; Model 2: OR = 1.25, 95%CI: 1.01—1.54) were positively associated with the risk of HUA in the unadjusted model and after adjusting for age, duration of T2DM, FPG, FINS, HOMA-β, AST, TG, HDL-C and eGFR (Model 2). After further adjusting for BMI in Model 3 and WC in Model 4, each SD increase in HC was still associated with a significantly increased risk of HUA (Model 3: OR = 1.36, 95%CI: 1.07—1.74; Model 4: OR = 1.51, 95%CI: 1.06—2.14), while other anthropometric indices showed no significant associations.

**Table 3:** Associations between different anthropometric indices and the risk of HUA in the middle-aged group (*n* = 791)

	**Model 1**^**a**^		**Model 2**^**b**^		**Model 3**^**c**^		**Model 4**^**d**^	
**Variable**	**OR (95%CI)**	***P* value**	**OR (95%CI)**	***P* value**	**OR (95%CI)**	***P* value**	**OR (95%CI)**	***P* value**
BMI	1.10 (1.05–1.17)	< 0.001	1.07 (1.01–1.14)	0.032				
Each SD increase in WC	1.39 (1.16–1.65)	< 0.001	1.27 (1.04–1.56)	0.022	1.18 (0.91–1.53)	0.221		
Each SD increase in HC	1.48 (1.23–1.78)	< 0.001	1.40 (1.14–1.72)	0.001	1.36 (1.07–1.74)	0.014	1.51 (1.06–2.14)	0.022
Each SD increase in WHR	1.05 (0.88–1.26)	0.594	0.94 (0.76–1.17)	0.589	0.85 (0.68–1.08)	0.184		
Each SD increase in WHtR	1.36 (1.14–1.63)	0.001	1.25 (1.01–1.54)	0.041	1.13 (0.86–1.49)	0.377		

*Abbreviations*: *HUA* Hyperuricemia, *BMI* Body mass index; *WC* Waist circumference, *HC* Hip circumference, *WHR* Waist-to-hip ratio, *WHtR* Waist-to-height ratio, *OR* Odds ratio, *CI* Confidence interval

^a^Model 1: Crude model

^b^Model 2: Adjusted for age, duration of T2DM, fasting plasma glucose, fast insulin, HOMA-β, aspartate aminotransferase, triglycerides, high-density lipoprotein cholesterol and estimated glomerular filtration rate

^c^Model 3: Further adjusted for BMI

^d^Model 4: Further adjusted for WC

In the older group (Table 4), the BMI, and each SD increase in WC, HC, and WHtR were also positively associated with the risk of HUA in Model 1 and Model 2 (all *P* < 0.05). However, after further adjusting for BMI in Model 3 and WC in Model 4, there was no association between HC and risk of HUA (all *P* > 0.05).

**Table 4:** Associations between different anthropometric indices and the risk of HUA in the older group (*n* = 656)

	**Model 1**^**a**^		**Model 2**^**b**^		**Model 3**^**c**^		**Model 4**^**d**^	
**Variable**	**OR (95%CI)**	***P* value**	**OR (95%CI)**	***P* value**	**OR (95%CI)**	***P* value**	**OR (95%CI)**	***P* value**
BMI	1.12 (1.05–1.18)	< 0.001	1.11 (1.04–1.18)	0.003				
Each SD increase in WC	1.41 (1.16–1.72)	< 0.001	1.30 (1.05–1.62)	0.018	1.11 (1.85–1.46)	0.430		
Each SD increase in HC	1.37 (1.12–1.68)	0.002	1.29 (1.04–1.61)	0.023	1.12 (0.87–1.44)	0.382	1.09 (0.76–1.55)	0.651
Each SD increase in WHR	1.18 (0.98–1.41)	0.082	1.11 (0.89–1.38)	0.356	1.01 (0.80–1.23)	0.926		
Each SD increase in WHtR	1.39 (1.15–1.68)	0.001	1.32 (1.06–1.64)	0.012	1.14 (0.87–1.49)	0.341		

*Abbreviations*: *HUA* Hyperuricemia, *BMI* Body mass index, *WC* Waist circumference, *HC* Hip circumference, *WHR* Waist-to-hip ratio, *WHtR* Waist-to-height ratio, *OR* Odds ratio, *CI* Confidence interval

^a^Model 1: Crude model

^b^Model 2: Adjusted for age, duration of T2DM, fasting plasma glucose, fast insulin, HOMA-β, aspartate aminotransferase, triglycerides, high-density lipoprotein cholesterol and estimated glomerular filtration rate

^c^Model 3: Further adjusted for BMI

^d^Model 4: Further adjusted for WC

### The AUC and optimal cutoff value of HC

**Fig. 2:**
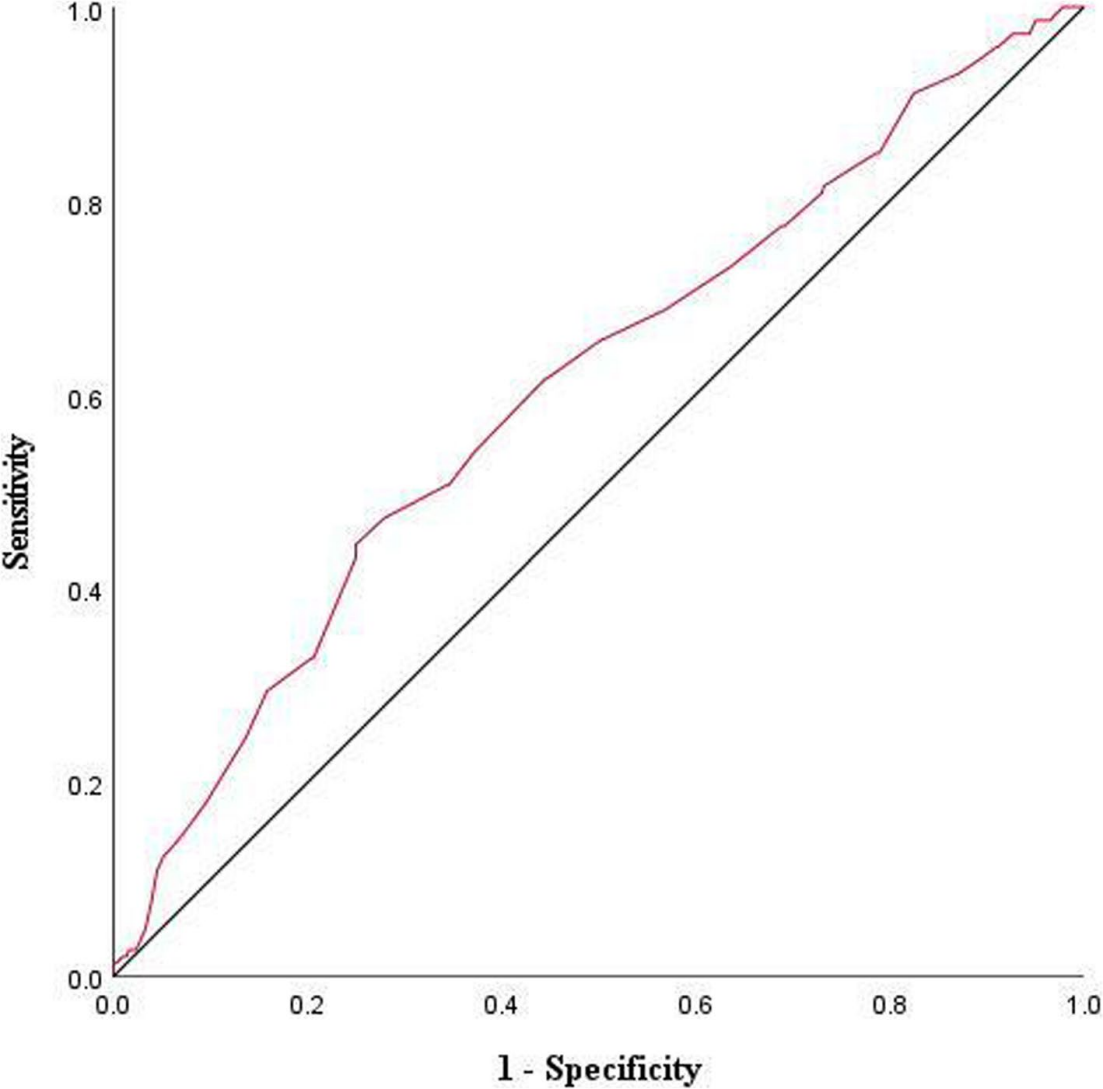
Receiver Operating Characteristic (ROC) Curve of Hip Circumference for Identifying Hyperuricemia in the Middle-Aged Male Patients with Type 2 Diabetes Mellitus. For detection of hyperuricemia, a cutoff value of 101.3 cm of hip circumference had a sensitivity of 44.5% and specificity of 75.0% with an area under the ROC curve of 0.607 (95%CI: 0.556—0.659; *P* < 0.001)

The ROC curve of HC for HUA in the middle-aged group was shown in Fig. 2. For detection of HUA, the optimal cutoff value was 101.3 cm of HC with an AUC of 0.607 (95%CI: 0.556—0.659; *P* < 0.001), giving a sensitivity of 44.5% and specificity of 75.0%.

## Discussion

In this study, BMI, WC, HC, and WHtR were positively associated with the risk of HUA in middle-aged and older male patients with T2DM. Moreover, HC also exhibited a significant association with a higher risk of HUA in the middle-aged group, independent of BMI and WC. And importantly, HC > 101.3 cm can be considered a potential predictor in HUA screening in middle-aged male patients with T2DM.

In our study, the prevalence of HUA in middle-aged and older male patients with T2DM was in line with the previous findings that HUA is more common in patients with T2DM than in middle-aged and older Chinese men general (19.3% vs. 7.8%) [17, 25, 26]. Some studies have demonstrated the association between high UA and insulin resistance (IR). An epidemiologic study [27] reported that high UA levels had a positive correlation with FINS levels. Regarding the mechanism involved, hyperinsulinemia may decrease UA clearance by the kidneys, because of the effects of insulin on proximal tubular urate transport of the kidney [28–30]. Similar findings were observed in this study that FINS levels were higher in HUA group than in non-HUA group.

The current study also showed a significant relationship between BMI and the risk of HUA. In line with our findings, a cross-sectional study conducted among T2DM patients in Southwestern Ethiopia exhibited that the study participants with obesity were 7.84 times more likely (OR = 7.84, 95%CI: 2.01—30.67) to develop HUA compared with underweight individuals [31]. In contrast, the prevalence of HUA was no significant difference between obese and non-obese Saudi Arabian adults T2DM patients (*P* = 0.791) [32]. These conflicting results may partly be explained by which BMI is an index of general obesity, unable to distinguish between central and peripheral fat.

Some studies suggested that the pattern of body fat distribution is more sensitive than BMI in predicting multiple diseases, since an accumulation of fat in abdominal viscera has been reported to be strongly related to IR independent of total adiposity [33]. As a marker of abdominal fat accumulation and visceral adiposity, higher WC and WHtR were positively associated with a higher risk of HUA in our population. This finding confirms the results from Huang’s study, which reported that male adults in the highest WC and WHtR groups had the highest HUA risks (OR = 1.76, 95%CI: 1.11—2.93]; OR = 2.57, 95%CI: 1.67—3.94; respectively) [34]. As for the mechanism, IR may play a mediator role in the relationship between obesity and HUA, as insulin has been identified as a cause of hyperuricemia by inhibiting uric acid excretion [35]. While there was no significant difference in WHR between HUA and non-HUA groups, it may be accounted that obese and lean individuals can have equal values in WHR [36]. And the current study has confirmed that WHR has limitations as a ratio index [37].

In addition to BMI, WC, and WHtR, many studies have raised the concerns on HC. In the present study, we also observed the positive association between HC and HUA in middle-aged male patients with T2DM but not in older, independent of BMI and WC, which was the highlight findings of this study. Moreover, the OR for HUA in the middle-aged group was significantly increased in HC (1.40) compared with WC (1.27), WHtR (1.25), and BMI (1.07), suggesting that subjects in this group seemed to be more sensitive to HC in terms of risk of HUA. Earlier studies performed by Yang, et al*.* and Eljaaly et al*.* also suggested that HC was positively related to the risk of HUA, although these two studies did not take the confounding effect of BMI and WC into consideration and did not perform further analysis in specific age groups [32, 38]. In addition, further ROC analysis yielded a cutoff value of 101.3 cm of HC for the detection of HUA in the middle-aged group.

The potential mechanisms that account for high prevalence of HUA with large HC are not clear, but there are several possibilities. First, larger HC may indicate a higher femoral and gluteal muscle mass in the middle-aged adults. Previous studies have shown some consistent findings regarding on the linkage between muscle mass and HUA. A cross-sectional study conducted among older adults aged from 50 to 85 years found that SUA levels were positively associated with muscle strength [39]. And another cross-sectional study performed on kidney transplant patients demonstrated that the UA levels were significantly associated with the muscle mass, fat-free mass, appendicular muscle mass, muscle mass index and appendicular muscle mass index [40], which was similar to our earlier findings in obese children and adolescents [41]. As is known to us, UA is the final oxidation product of purine catabolism. And 80% of purine compounds are derived from endogenous (nucleic acids and internal pool of purines) of the body [42]. Furthermore, muscle mass is considered as the largest source of purine in the body [43]. During the process of its growth, depletion of muscle cells or metabolism of adenosine triphosphate results in releasing plenty of nucleic acids and purines, and increased production of UA [42, 44]. Therefore, it is reasonable that an increase in muscle mass leads to an increase in serum UA level. Although previous studies suggested that adipose tissue is associated with UA secretion because of UA transporter URAT1 and abundant activity of xanthine oxidase in adipose tissue [7, 45]. In our opinion, this does not conflict with the notion that muscle affects uric acid production as a source of purine. Second, Tsushima et al. provided the evidence that muscle has higher xanthine oxidase activity in obese mice than in C57 mice, which may lead to an increase in the secretion of UA [45]. And it is worth noting that skeletal muscle mass is the main tissue target for insulin action and therefore a major site for IR [46]. It would be necessary to conduct experiments to elucidate the connection between UA and IR in muscle in the future study.

As for the inconsistent results of the relationship between HC and HUA in the middle-aged and older group, we hypothesized that the correlation between HC and muscle mass was lower in the older group than in the middle-aged group, because the older individuals have more loss and less synthesis of muscle mass occurring in aging [47]. According to the skeletal muscle mass measured by magnetic resonance imaging in a large and heterogeneous sample of 468 men and women, the skeletal muscle mass in 40–49 years old, 50–59 years old, 60–69 years old, and above 70-years old in men was 33.5 ± 5.5 kg, 31.4 ± 4.8 kg, 30.2 ± 3.1 kg, and 27.8 ± 3.4 kg, respectively [48].

### Strengths and limitations

The strengths of our study include the relatively large sample size of the associations between different anthropometric indices and HUA among middle-aged and older male patients with T2DM, the use of logistic regression model with BMI and WC further adjusted, and calculating the cutoff value of HC for HUA screening. To our best knowledge, this is the first study that showed the cutoff value of HC for identifying HUA in middle-aged male patients with T2DM.

Of course, our study also had some limitations. First, the single-center retrospective design has some limitations with regard to interpreting the causality of associations. Second, the WC and HC may not reflect actual abdominal obesity and gluteal muscle mass, respectively. Third, because the present study focused on the relationship between anthropometric indices and HUA, rather than lifestyle, the smoking and drinking status was not collected, which may confound the outcomes. Therefore, it would be planned to acquire the data such as the smoking and drinking status, and the actual amount and size of abdominal adipocyte and gluteal muscle mass measured using bioelectrical impedance analyses or dual energy X-ray absorptiometry in the following study. Last, as the predictive power of anthropometric measures for chronic disease risk is population-dependent and varies from race to race [11, 36, 49, 50], the findings in current study should be cautious to generalize to other ethnic groups.

## Conclusions

In this study, the findings indicated that HC was a stronger predictor of HUA in middle-aged but not in older male patients with T2DM, independent of BMI and WC. Considering the physical and economic burden of HUA, more attention should be paid to HC with the cutoff value of 101.3 cm in clinical practice to reduce the risk of HUA in middle-aged male patients with T2DM. Further prospective studies with more and larger sample sizes should be conducted to provide stronger evidence on this association and the cutoff value of HC.

## Data Availability

The data that support the findings of this study are available from the corresponding author upon reasonable request.

## Abbreviations

T2DM: Type 2 diabetes mellitus
WC: Waist circumference
HC: Hip circumference
BMI: Body mass index
WHR: Waist-to-hip ratio
WHtR: Waist-to-height ratio
HUA: Hyperuricemia
UA: Uric acid
BP: Blood pressure
TG: Triglycerides
HDL-C: High-density lipoprotein cholesterol
LDL-C: Low-density lipoprotein cholesterol
TC: Total cholesterol
FPG: Fasting plasma glucose
ALT: Alanine aminotransferase
AST: Aspartate aminotransferase
FINS: Fasting insulin
HOMA: Homeostasis model assessment
HOMA-IR: Homeostasis model assessment for insulin resistance
HOMA-β: Homeostasis model assessment for β-cell function
eGFR: Estimated glomerular filtration rate
SD: Standard deviation
IQR: Interquartile ranges
OR: Odds Ratio
CI: Confidence interval
ROC: Receiver operating characteristic
AUC: Area under receiver operating characteristic curves
IR: Insulin resistance

## References

[1] Sun H, Saeedi P, Karuranga S, Pinkepank M, Ogurtsova K, Duncan BB, Stein C, Basit A, Chan JCN, Mbanya JC (2022). IDF Diabetes Atlas: Global, regional and country-level diabetes prevalence estimates for 2021 and projections for 2045. Diabetes Res Clin Pract.

[2] Hu C, Jia W (2018). Diabetes in China: epidemiology and genetic risk factors and their clinical utility in personalized medication. Diabetes.

[3] Doria A, Galecki AT, Spino C, Pop-Busui R, Cherney DZ, Lingvay I, Parsa A, Rossing P, Sigal RJ, Afkarian M (2020). Serum urate lowering with allopurinol and kidney function in type 1 diabetes. N Engl J Med.

[4] Li B, Chen L, Hu X, Tan T, Yang J, Bao W, Rong S (2023). Association of serum uric acid with all-cause and cardiovascular mortality in diabetes. Diabetes Care.

[5] Li S, Cheng J, Cui L, Gurol ME, Bhatt DL, Fonarow GC, Benjamin EJ, Xing A, Xia Y, Wu S, Gao X (2019). Cohort Study of Repeated Measurements of Serum Urate and Risk of Incident Atrial Fibrillation. J Am Heart Assoc.

[6] Kuwabara M, Niwa K, Nishi Y, Mizuno A, Asano T, Masuda K, Komatsu I, Yamazoe M, Takahashi O, Hisatome I (2014). Relationship between serum uric acid levels and hypertension among Japanese individuals not treated for hyperuricemia and hypertension. Hypertens Res.

[7] Sautin YY, Nakagawa T, Zharikov S, Johnson RJ (2007). Adverse effects of the classic antioxidant uric acid in adipocytes: NADPH oxidase-mediated oxidative/nitrosative stress. Am J Physiol Cell Physiol.

[8] Zhang N, Chang Y, Guo X, Chen Y, Ye N, Sun Y (2016). A Body Shape Index and Body Roundness Index: Two new body indices for detecting association between obesity and hyperuricemia in rural area of China. Eur J Intern Med.

[9] Kuo KL, Chen HM, Hsiao SH, Chu D, Huang SJ, Huang KC, Huang CY (2021). The relationship between anthropometric factors and hyperuricemia in adolescent athletes. Obes Res Clin Pract.

[10] Tao M, Ma X, Pi X, Shi Y, Tang L, Hu Y, Chen H, Zhou X, Du L, Chi Y (2021). Prevalence and related factors of hyperuricaemia in Shanghai adult women of different ages: a multicentre and cross-sectional study. BMJ Open.

[11] Esmaillzadeh A, Mirmiran P, Moeini SH, Azizi F (2006). Larger hip circumference independently contributed to reduced metabolic risks in Tehranian adult women. Int J Cardiol.

[12] Jung KJ, Kimm H, Yun JE, Jee SH (2013). Thigh circumference and diabetes: obesity as a potential effect modifier. J Epidemiol.

[13] Heitmann BL, Frederiksen P (2009). Thigh circumference and risk of heart disease and premature death: prospective cohort study. BMJ.

[14] Shi J, Yang Z, Niu Y, Zhang W, Lin N, Li X, Zhang H, Gu H, Wen J, Ning G (2020). Large thigh circumference is associated with lower blood pressure in overweight and obese individuals: a community-based study. Endocr Connect.

[15] Yusuf S, Hawken S, Ounpuu S, Bautista L, Franzosi MG, Commerford P, Lang CC, Rumboldt Z, Onen CL, Lisheng L (2005). Obesity and the risk of myocardial infarction in 27,000 participants from 52 countries: a case-control study. Lancet.

[16] Chen GC, Arthur R, Iyengar NM, Kamensky V, Xue X, Wassertheil-Smoller S, Allison MA, Shadyab AH, Wild RA, Sun Y (2019). Association between regional body fat and cardiovascular disease risk among postmenopausal women with normal body mass index. Eur Heart J.

[17] Song P, Wang H, Xia W, Chang X, Wang M, An L (2018). Prevalence and correlates of hyperuricemia in the middle-aged and older adults in China. Sci Rep.

[18] Dong X, Zhang H, Wang F, Liu X, Yang K, Tu R, Wei M, Wang L, Mao Z, Zhang G, Wang C (2020). Epidemiology and prevalence of hyperuricemia among men and women in Chinese rural population: The Henan Rural Cohort Study. Mod Rheumatol.

[19] Yu S, Yang H, Guo X, Zhang X, Zhou Y, Ou Q, Zheng L, Sun Y (2016). Prevalence of hyperuricemia and its correlates in rural Northeast Chinese population: from lifestyle risk factors to metabolic comorbidities. Clin Rheumatol.

[20] Diagnosis and classification of diabetes mellitus. Diabetes Care 2013, 36 Suppl 1:S67-74. 10.2337/dc13-S067 PMC3537273 23264425

[21] Iseki K, Ikemiya Y, Inoue T, Iseki C, Kinjo K, Takishita S (2004). Significance of hyperuricemia as a risk factor for developing ESRD in a screened cohort. Am J Kidney Dis.

[22] Wallace TM, Levy JC, Matthews DR (2004). Use and abuse of HOMA modeling. Diabetes Care.

[23] Matthews DR, Hosker JP, Rudenski AS, Naylor BA, Treacher DF, Turner RC (1985). Homeostasis model assessment: insulin resistance and beta-cell function from fasting plasma glucose and insulin concentrations in man. Diabetologia.

[24] Ma YC, Zuo L, Chen JH, Luo Q, Yu XQ, Li Y, Xu JS, Huang SM, Wang LN, Huang W (2006). Modified glomerular filtration rate estimating equation for Chinese patients with chronic kidney disease. J Am Soc Nephrol.

[25] Woyesa SB, Hirigo AT, Wube TB (2017). Hyperuricemia and metabolic syndrome in type 2 diabetes mellitus patients at Hawassa university comprehensive specialized hospital South West Ethiopia. BMC Endocr Disord.

[26] Wang J, Chen RP, Lei L, Song QQ, Zhang RY, Li YB, Yang C, Lin SD, Chen LS, Wang YL (2013). Prevalence and determinants of hyperuricemia in type 2 diabetes mellitus patients with central obesity in Guangdong Province in China. Asia Pac J Clin Nutr.

[27] Yoo TW, Sung KC, Shin HS, Kim BJ, Kim BS, Kang JH, Lee MH, Park JR, Kim H, Rhee EJ (2005). Relationship between serum uric acid concentration and insulin resistance and metabolic syndrome. Circ J.

[28] Quiñones Galvan A, Natali A, Baldi S, Frascerra S, Sanna G, Ciociaro D, Ferrannini E (1995). Effect of insulin on uric acid excretion in humans. Am J Physiol.

[29] Muscelli E, Natali A, Bianchi S, Bigazzi R, Galvan AQ, Sironi AM, Frascerra S, Ciociaro D, Ferrannini E (1996). Effect of insulin on renal sodium and uric acid handling in essential hypertension. Am J Hypertens.

[30] Reaven GM (1997). The kidney: an unwilling accomplice in syndrome X. Am J Kidney Dis.

[31] Arersa KK, Wondimnew T, Welde M, Husen TM (2020). Prevalence and determinants of hyperuricemia in type 2 diabetes mellitus patients attending Jimma Medical Center, Southwestern Ethiopia, 2019. Diabetes Metab Syndr Obes.

[32] Eljaaly Z, Mujammami M, Nawaz SS, Rafiullah M, Siddiqui K (2021). Risk predictors of high uric acid levels among patients with type-2 diabetes. Diabetes Metab Syndr Obes.

[33] Gastaldelli A, Sironi AM, Ciociaro D, Positano V, Buzzigoli E, Giannessi D, Lombardi M, Mari A, Ferrannini E (2005). Visceral fat and beta cell function in non-diabetic humans. Diabetologia.

[34] Huang ZP, Huang BX, Zhang H, Zhu MF, Zhu HL (2019). Waist-to-Height ratio is a better predictor of hyperuricemia than body mass index and waist circumference in Chinese. Ann Nutr Metab.

[35] Li C, Hsieh MC, Chang SJ (2013). Metabolic syndrome, diabetes, and hyperuricemia. Curr Opin Rheumatol.

[36] Molarius A, Seidell JC (1998). Selection of anthropometric indicators for classification of abdominal fatness–a critical review. Int J Obes Relat Metab Disord.

[37] Allison DB, Paultre F, Goran MI, Poehlman ET, Heymsfield SB (1995). Statistical considerations regarding the use of ratios to adjust data. Int J Obes Relat Metab Disord.

[38] Yang H, Liu C, Jin C, Yu R, Ding L, Mu L (2021). Neck Circumference is associated with hyperuricemia in women with polycystic ovary syndrome. Front Endocrinol (Lausanne).

[39] Nahas PC, Rossato LT, de Branco FMS, Azeredo CM, Rinaldi AEM, de Oliveira EP (2021). Serum uric acid is positively associated with muscle strength in older men and women: Findings from NHANES 1999–2002. Clin Nutr.

[40] Floriano JP, Nahas PC, de Branco FMS, Dos Reis AS, Rossato LT, Santos HO, Limirio LS, Ferreira-Filho SR, de Oliveira EP: Serum Uric Acid Is Positively Associated with Muscle Mass and Strength, but Not with Functional Capacity, in Kidney Transplant Patients. Nutrients 2020, 12. 10.3390/nu12082390 PMC7469022 32785016

[41] Xie L, Mo PKH, Tang Q, Zhao X, Zhao X, Cai W, Feng Y, Niu Y (2022). Skeletal muscle mass has stronger association with the risk of hyperuricemia than body fat mass in obese children and adolescents. Front Nutr.

[42] Maiuolo J, Oppedisano F, Gratteri S, Muscoli C, Mollace V (2016). Regulation of uric acid metabolism and excretion. Int J Cardiol.

[43] Lowenstein JM (1972). Ammonia production in muscle and other tissues: the purine nucleotide cycle. Physiol Rev.

[44] Hammouda O, Chtourou H, Chaouachi A, Chahed H, Ferchichi S, Kallel C, Chamari K, Souissi N (2012). Effect of short-term maximal exercise on biochemical markers of muscle damage, total antioxidant status, and homocysteine levels in football players. Asian J Sports Med.

[45] Tsushima Y, Nishizawa H, Tochino Y, Nakatsuji H, Sekimoto R, Nagao H, Shirakura T, Kato K, Imaizumi K, Takahashi H (2013). Uric acid secretion from adipose tissue and its increase in obesity. J Biol Chem.

[46] Yki-Järvinen H, Koivisto VA, Karonen SL (1985). Influence of body composition on insulin clearance. Clin Physiol.

[47] Batsis JA, Villareal DT (2018). Sarcopenic obesity in older adults: aetiology, epidemiology and treatment strategies. Nat Rev Endocrinol.

[48] Janssen I, Heymsfield SB, Wang ZM, Ross R (1985). Skeletal muscle mass and distribution in 468 men and women aged 18–88 yr. J Appl Physiol.

[49] Gallagher D, Visser M, Sepúlveda D, Pierson RN, Harris T, Heymsfield SB (1996). How useful is body mass index for comparison of body fatness across age, sex, and ethnic groups?. Am J Epidemiol.

[50] Lear SA, Chen MM, Frohlich JJ, Birmingham CL (2002). The relationship between waist circumference and metabolic risk factors: cohorts of European and Chinese descent. Metabolism.

